# Environmental exposure and sensitization patterns in a Swiss alpine pediatric cohort^☆^

**DOI:** 10.1016/j.waojou.2023.100847

**Published:** 2023-12-03

**Authors:** Karin B. Fieten, José M. Maya-Manzano, Beate Rückert, Joana Candeias, Gudrun Pusch, CK-CARE Study Group, Peter Schmid-Grendelmeier, Roger Lauener, Thomas Bieber, Marie-Charlotte Brüggen, Ellen Renner, Claudia Traidl-Hoffmann, Cezmi Akdis, Jeroen Buters, Cezmi A. Akdis, Claudia Traidl-Hoffmann

**Affiliations:** a Hochgebirgsklinik Davos, Davos, Switzerland; b Swiss Institute for Allergy and Asthma Research (SIAF), University of Zürich, Davos, Switzerland; c Christine Kühne Center for Allergy Research and Education (CK-CARE), Davos, Switzerland; d Center of Allergy & Environment (ZAUM), Member of the German Center for Lung Research (DZL), Technische Universität München/Helmholtz Center, Munich, Germany; e Department of Plant Biology, Ecology and Earth Sciences, University of Extremadura, Badajoz, Spain; f Environmental Medicine, Faculty of Medicine, University of Augsburg, Augsburg, Germany; g Institute of Environmental Medicine, Helmholtz Center Munich - German Research Center for Environmental Health, Augsburg, Germany

**Keywords:** Allergic sensitization, Alpine, Environment, Exposure, Pollution

## Abstract

**Background:**

The level of environmental exposure throughout life may contribute to the prevalence of allergic sensitization and allergic disease. The alpine climate has been considered a healthy climate with little allergen exposure and pollution. We conducted a cross-sectional study to investigate local environmental exposure and concomitant prevalence of allergic sensitization among local school children born and raised in an alpine environment.

**Methods:**

Clinical and demographic data were collected with a questionnaire. Allergen content was assessed in residential settled dust samples, lifetime exposure to pollen and air pollution was calculated using data from national pollen and air pollution monitoring stations, and the allergic sensitization profile was determined with component resolved diagnostics (ISAC®). Univariate and multivariate regression models were used to estimate the relation between exposure and sensitization.

**Results:**

In a cohort of children born and raised in an alpine environment, sensitization to aeroallergens is quite common (38%), especially to grass (33%) and cat (16%). House dust mite allergen was detected in up to 38% of residential dust samples, but sensitization to HDM was low (2.5%). Pollutant levels were low, but an increasing trend was observed in the amount of ozone and PM_10_. Living close to a busy road was associated with increased odds OR (95% CI) for being sensitized to any allergen 2.7 (1.0–7.2), to outdoor allergens 2.8 (1.1–7.1) and being sensitized plus reporting symptoms of rhinoconjunctivitis 4.4 (1.3–14.8) and asthma 5.5 (1.4–21). Indoor living conditions, including the presence of visible mold, increased the odds of being sensitized to indoor allergens (1.9 (1.1–3.2) and being sensitized plus reporting symptoms of rhinoconjunctivitis 1.9 (1.0–3.6) and asthma 2.1 (1.0–4.1).

**Conclusion:**

In a healthy alpine environment, pollution might still be an important factor contributing to allergic sensitization.

## Introduction

The prevalence of allergic sensitizations and allergic diseases have been increasing over the last decades.^1^, ^2^, ^3^ Environmental exposure throughout life, or exposome, likely contributes to this increase.^4^ The exposome consists of the general external environment (climate, biodiversity, urban-rural, social and economic factors), the specific external environment (diet, physical activity, allergens, microbes, pollutants) and the host dependent internal environment (age, metabolic factors, inflammation, oxidative stress).^5^^,^^6^ The exposome is able to modulate the development of allergic sensitization.^7^ There is considerable variation in allergic prevalence between countries, within countries and even within cities, indicating the importance of local environmental characteristics.^2^ Timing and duration of environmental exposure are important determinants of the development and progression of allergic diseases.^8^

Several significant risk factors for the development of allergic disease have been identified, such as indoor allergen exposure and the abundance and diversity of microbial exposure during the first years of life.^4^^,^^9^^,^^10^ Indoor allergen exposure levels can be influenced by several environmental, socioeconomic and demographic factors and previous studies have found large differences in allergen exposure between groups.^8^ The abundance and diversity of microbial exposure during the first years of life have been linked with the risk of allergic sensitization.^9^ Exposure to traffic related air pollution (TRAP) has also been linked to allergic sensitization and the development of allergic disease.^11^, ^12^, ^13^ However, there is a complex interplay between the different exposome indoor and outdoor environmental factors, host factors, timing, and location of exposure and subsequent development or exacerbation of allergic disease and no single factor has been identified that dominates disease development in every individual.^8^

The alpine climate has been considered a healthy climate with little exposure to allergens and little pollution. The Swiss municipality Davos is located at 1560 m altitude in the Alps and its climate is characterized by low presence of aeroallergens such as house dust mites (HDM), pollen and fungal spores and low amounts of TRAP such as particulate matter (PM10), nitrogen oxides (NOx) and ozone (O3).^14^ The pollen season in Davos can be short and clinically relevant pollen concentrations are sometimes observed only a few days per year. It has been generally accepted that with increasing altitude and decreasing humidity, HDM allergen concentration decreases. So far, few studies have investigated local environmental exposure in the alpine environment together with allergic sensitizations in its local population.

This cross-sectional study aims to investigate the relation between environmental exposure and prevalence of allergic sensitization among school children born and raised in the Swiss alpine municipality Davos.

## Methods

### Study design and participants

We conducted a cross-sectional study in the alpine municipality Davos, Switzerland at 1560 m altitude. School children aged 5–9 years attending school (kindergarten to second grade) in the municipality of Davos were asked to participate in the study. Exclusion criteria were not born in Davos or having been away from Davos for longer than 6 months. Participants were included in the study between October 2017 and May 2019. Written informed consent was obtained from the legal guardians.

### Clinical and demographic characteristics

Clinical and demographic data was collected with the use of a questionnaire, filled out by the legal guardians of the child. The questionnaire included questions on sex, age, socioeconomic status and others. Data was collected on the birth of the child, breastfeeding, number of siblings, general health of the child, use of antibiotics, vaccination status, day care attendance, and time spent outdoors. Residential characteristics such as type of residence, total living area, number of persons living in the home, proximity to a busy road, and type of heating were collected. Furthermore, data was collected on exposure to smoke, exposure to animals, presence of pets in the home, washing and changing of bed linens, and family history of atopy.

Presence of atopic disease was assessed with the ISAAC questions for asthma, rhinitis, atopic dermatitis and food allergy. Furthermore doctor-diagnosed allergies or atopic diseases were recorded, as well presence of allergies and intolerances by recording use of antihistamines, previous allergy tests, possession of an anaphylaxis emergency kit or previous immunotherapy, subcutaneous immunotherapy/sublingual immunotherapy (SCIT/SLIT).

### Allergen content in residential settled dust samples

Individual house dust samples were collected by study participants using a commercially available DUSTREAM Collector (Indoor Biotechnologies, Charlottesville, VA, USA) attached to a household vacuum cleaner. Participants were asked to vacuum the mattress, pillow and floor of the bedroom where the child sleeps, as well as the couch and playroom or other rooms where the child spends much time. Dust extracts were prepared by removing large particles/hairs and dissolving 100 mg of dust in 2 ml of phosphate-buffered saline pH 7.4, 0.05% Tween-20. Samples <10 mg of dust were not considered for further analysis. Proteins were extracted by incubation on a roll mixer for 2 h at room temperature following a centrifugation step at 13,000×*g* for 20 min. Supernatants were stored at −80 °C until further processing. For allergen content analysis, the dust extracts were thawed and centrifuged again. Supernatants were diluted 1:10, 1:100 and 1:10,000 and examined in a Multiplex Array for Indoor Allergens (MARIA, Indoor Biotechnologies, Charlottesville, VA, USA) using xMAP Technology (Luminex, Austin, TX, USA). The array uses fluorescently labeled beads conjugated to monoclonal antibodies specific for purified allergen molecules. The allergen concentration of Der p 1, Der p 2 and Der f 1 allergens (house dust mite allergens), Mus m 1 (mouse allergen), Asp f 1 and Alt a 1 (*Aspergillus* and *Alternaria* mold allergen) as well as Ara h 6 (peanut allergen) was investigated. A 12-point standard curve executed in duplicates was used to quantify the results using the 1:10 dust extract dilution. Additionally, quality controls provided with the test kit were applied. Measurements of fluorescence were performed in the Bio-Plex 200 System (BioRad Laboratories, Hercules, CA, USA). For samples below the lower limit of detection no quantification was calculated, but a present/not-present was calculated when a sample gave an OD above 3 S.D. of the blank.

### Exposure to weather, pollen, and pollution

Davos is located at the bottom of a valley. Predominant wind directions for this city are 0–45° and 180–225°, reaching 1022 mm as annual rate of precipitation and having an average mean temperature of 3.5 °C.^14^

Meteoswiss operates a national pollen monitoring network and data from the station (PDS) at 1587 m altitude at Davos Wolfgang was collected.^14^ Daily pollen data (pollen grains/m^3^) for hazel, alder, birch, plane, grass, plantain, mugwort, and ambrosia were collected for each child from the month of birth until blood was collected to calculate cumulative exposure measures.

The federal office for the environment collects data on air pollution through the national air pollution monitoring network.^15^ Daily pollution measurements (μg/m³) regarding NO, NO_2_, NOx, O_3_, PM_2.5_ and PM_10_ were collected from the measurement stations DPR 46.79,654 N, 9.82240 E, 1556.8 m (until December 2017) and DBP 46.79,304 N, 9.82088 E, 1556.5 m (from December 2017) in Davos Platz. Welch's *t*-test showed no differences between the stations and data was combined for the whole time period.

### Sensitization profile

Study participants provided a capillary or venous blood sample. Blood samples were obtained from the fingertip, incubated at room temperature and centrifugated for 5–10 min at 4350 rpm. Serum was separated and stored at −20 °C until further analysis.

Analysis of sera for specific IgE to single purified allergen components was done by means of the allergen multiplex array ImmunoCAP ISAC (Thermo Fisher Scientific, Uppsala, Sweden). According to the manufacturer's protocol, the test was performed with 30 μl of serum (Protocol No. 20-01-02-6). The resulting fluorescent signals were measured with a confocal laser scanner (LuxScan-10K, CapitalBio, Beijing, China). Data were analyzed using Phadia Microarray Image Analyzer (MIA) software and transformed into semi-quantitative ISAC Standardized Units (ISU). Specific IgE values ≥ 0.3 ISU were considered positive. Participants with a positive value to any of the 112 allergens on the ImmunoCAP ISAC were considered sensitized.

### Statistical analysis

Descriptive statistics were used to describe sensitization patterns of the study participants. Univariate regression models were constructed to assess correlations between specific IgE values (Der f 1, Der p 1, Der p 2, Der f 1, Mus m 1, Alt a 1, Asp f 1, and Ara h 6) and corresponding allergen component concentrations in residential dust and cumulative pollen exposure (Amb a 1, Aln g 1, Bet v 1, Bet v 2, Cyn d 1, Phl p 1–12, Cor a 1.0101, Plane a 1–3, Pla l 1 Art v 1 Art v 3). Time series analysis was conducted for air pollutants NO, NO_x_, NO_2_, O_3_, PM_10_ and pollen with an Annual Pollen Integral of APIn >100 pollen ∗ day/m3 for the studied period (*Alnus, Corylus, Betula*, Poaceae and *Plantago*) with LOESS smoothing to determine trends. Principal component analysis was used to group exposure variables from the questionnaire. Sensitization to aeroallergens was divided into indoor allergens (dog, cat, mouse, HDM, cockroach, *Alternaria*) and outdoor (grass, tree, weed, horse) allergens.

Multiple logistic regression analysis was used to estimate the relation between exposure and sensitization, corrected for the confounding factors family history of atopy and sex. Statistical analyses were performed with IBM SPSS statistics for Windows version 23 and GraphPad Prism 7 for Windows (GraphPad Software, Inc, La Jolla, CA, USA). Pollen and pollution graphs and the statistical analysis were carried out with R aeRobiology package (R Core team 2014). Two-sided p values < 0.05 were considered statistically significant. No multiplicity adjustment was used.

## Results

### Study participants

**Fig. 1:**
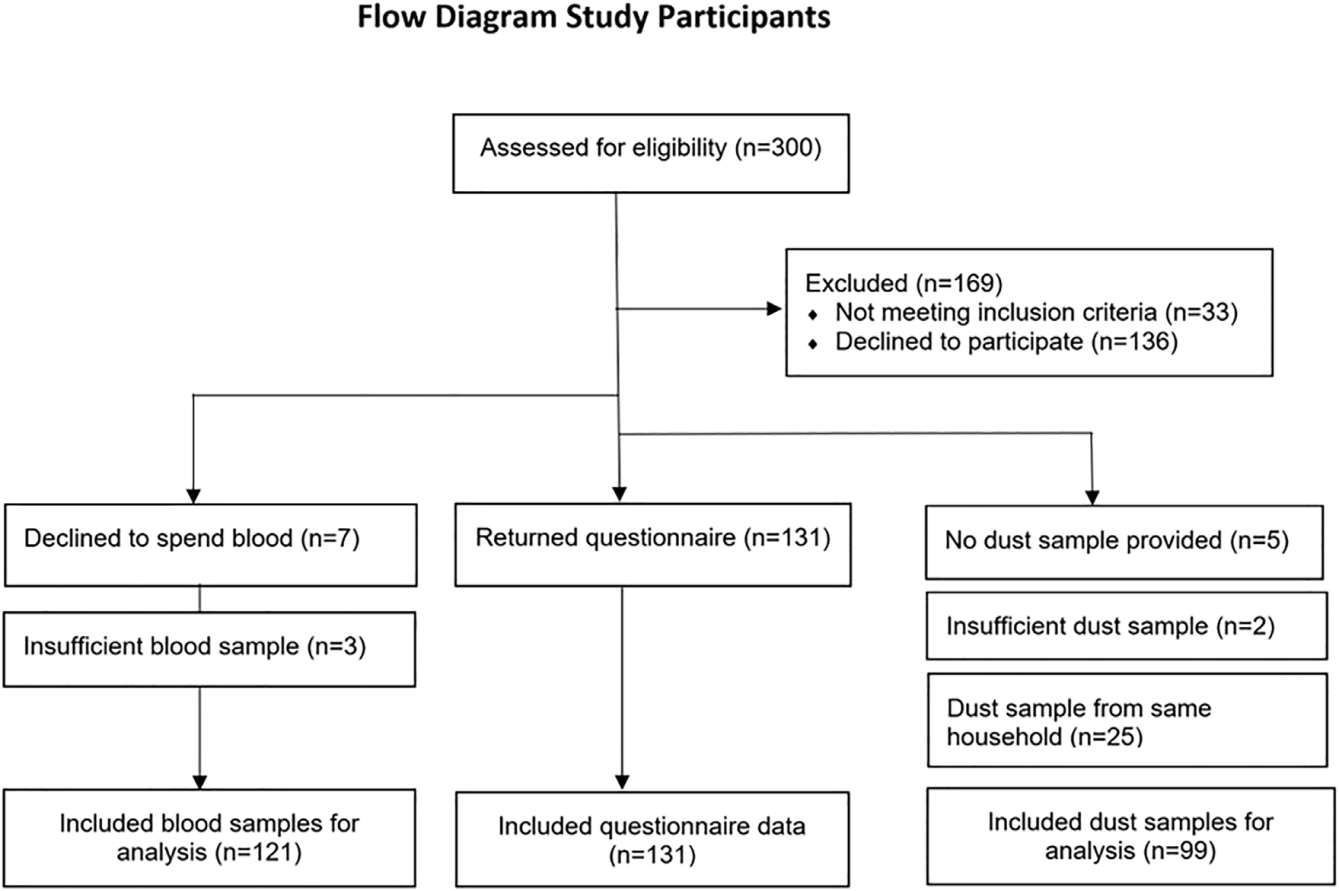
Flowchart of study participants and provided blood and dust samples

A total of 131 children participated in the study, of whom 121 also donated blood (Fig. 1). In total 99 dust samples were analyzed, some study participants did not provide a sample, two samples were excluded because not enough dust was provided, 25 samples were excluded because their siblings participated in the study and the sample would represent the same household.

Table 1 shows the demographic characteristics of the study population. The mean age of the study participants was 6.8 years and half were girls. Most had at least one sibling, a third were born with c-section. Median breastfeeding time was 8 months. Almost half had pets in the home and a quarter reported living close to a busy road. Almost two thirds had regular exposure to animals. Half of the study population spent more than 2 h outdoors each day. Almost two thirds had atopic family members and one third reported current or previous visible mold in the home. Socio-economic position was calculated using educational level of both parents, number of persons in the household, number of rooms in the house and marital status (Supplementary Table 2).

**Table 1:** Clinical and demographic data of study participants.

Characteristic	Study participants (n = 131)
Age (years) mean(SD)	6.8 (1.3)
Sex N(% female)	64(49 %)
Weight (kg) mean(SD)	23 (5.5)
Height (cm) mean(SD)	122 (9.8)
Number of siblings	
none	18 (14 %)
1	60 (46 %)
2	44 (34 %)
>3	9 (7 %)
Born with c-section	46 (35 %)
Ever breastfed	115 (88 %)
Exclusively breastfed	96 (73 %)
Total breastfeeding time in months median(IQR)	8 (6)
Vaccinated according to plan	105 (80 %)
Use of antibiotics in first year of life	22 (17 %)
Type of residence (apartment)	78 (60 %)
Living or having lived on a farm with livestock	18 (14 %)
Living close to a busy road	33 (25 %)
Number of persons in the house (median, IQR)	4 (1)
Open fireplace	13 (10 %)
Presence of balcony	99 (76 %)
Daily time spent outdoors (>2 h)	64 (49 %)
Day care attendance	104 (79 %)
Environmental changes to prevent allergies	9 (7 %)
Visible presence of molds in the home	41 (31 %)
Exposure to smoke	37 (28 %)
Pets in the home	57 (43 %)
Regular exposure to animals	82 (63 %)
Frequent visits to a cow shed (>1x month)	34 (26 %)
Weekly change of bed linen	15 (12 %)
Washing temperature bed linen over 60° ˚C	110 (84 %)
Indoor exposure to more than 2 allergens	42 (42 %)
Atopic family members	81 (62 %)
Anaphylaxis rescue set	11 (8 %)
Sensitized according to ISAC (%)	51 (42 %)
Self-reported allergic reaction (%)	34 (26 %)
Self-reported asthma	31 (24 %)
Self-reported atopic dermatitis	19 (15 %)
Self-reported rhinitis	28 (22 %)
Self-reported food allergy	12 (9 %)
Parents born elsewhere	
One parent	32 (24 %)
Both parents	21 (16 %)
Socio-economic position	
Low	24 (18 %)
Middle	73 (56 %)
High	34 (26 %)

SD = standard deviation, IQR = inter quartile range, ISAC = Immuno-Solid phase Allergy Chip

### Exposure

#### Indoor allergens

**Fig. 2:**
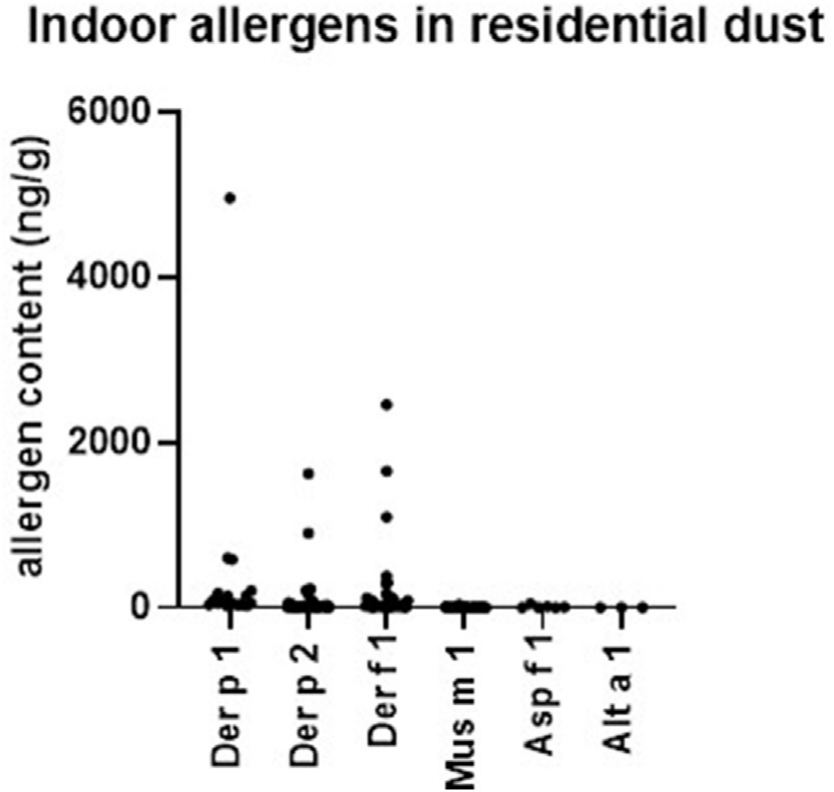
Indoor allergen concentrations (ng/g) measured in residential dust. Detection rates of Der p 1 was 17 %, Der p 2 37 %, Der f 1 38 %, Mus m 1 50 %, Asp f 1 6 %, Alt a 1 3 %. Dots represent individual measurements (n = 99)

We found detectable levels of the house dust mite allergen Der p 1 in 17%, Der p 2 in 37%, Der f 1 in 38%, Mus m 1 in 50%, Asp f 1 in 6%, Alt a 1 in 3% and Ara h 6 in 99% of households (Supplementary Figure 1). The median of detectable HDM samples was 74 (16–4960) ng/g for Der p 1, the median of detectable Der p 2 was 18 (2.1–1620), and the median for Der f 1 was 29 (7–2460) ng/g. For mouse allergen Mus m 1, the median of positive samples was 2.7 (0.4–36) ng/g. The mold allergens Alt a 1 and Asp f 1 had a median of 3.3 (2.4–3.4) ng/g (3 positive samples) and a median of 6 ng/g (3.3–50) (6 positive samples) (Fig. 2). For peanut allergen Ara h 6, all samples but 1 were positive and the median was 432 (13–11,500) ng/g (Supplementary Figure 2).

Exposure to multiple allergens was present in the majority of households (Supplementary Figure 3). In 27% of households the peanut allergen Ara h 6 was the only detected allergen. In 31% of households 2 allergens were detected, in 18% of households 3 allergens were detected, in 14% of households 4 different allergens were detected, in 8% of households 5 different allergens were detected, in 2% of households 6 different allergens were detected. All but 1 household with Der p 1 present also was positive for Der p 2, 78% of samples positive for Der p 2 were also positive for Der f 1.

#### Pollen

**Fig. 3:**
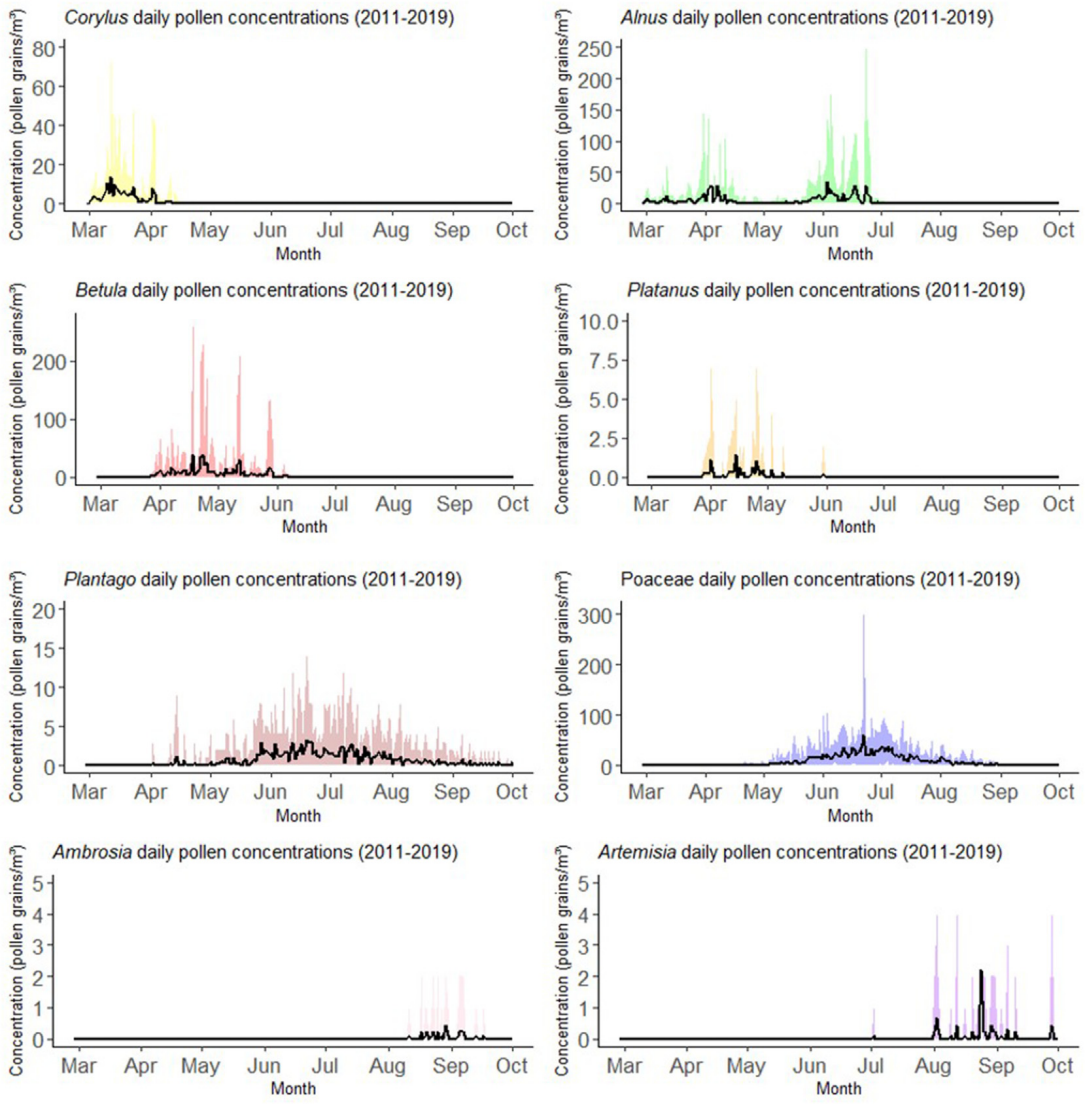
Daily pollen concentrations of the most common pollen. The black line shows the mean along the period (2011–2019) and the colored areas show the ranges for the concentration values during the studied period

Hazel pollen were mainly detected in March to April, alder pollen from March until June, birch pollen in April and May, plane in April and May, grasses from May until August, plantain from May until August, mugwort and ambrosia were sporadically detected in August and September (Fig. 3). The relative abundance of pollen in the air is displayed in Supplementary Figure 4. average yearly exposure in the period from 2011 to 2019 was for alder 717 ± 425 (pollen ∗ day/m^3^), hazel 147 ± 114, birch 564 ± 3774, plane 11 ± 51, grass 1578 ± 3418, plantain 136 ± 29, mugwort 7 ± 8 and ambrosia 3 ± 2 (Supplementary Figure 5). Cumulative exposure measures were calculated for hazel (median(IQR)) 1183(414), birch (median(IQR)) 3622(1304), plane (median(IQR)) 69(12), grass (median(IQR)) 12,182(2751), plantain (median(IQR)) 978(201), mugwort (median(IQR)) 59(35), ambrosia (median(IQR)) 20(7), and alder median(IQR) 5385(1540). Significant increasing time trends were observed for herbs and Plantago, Poaceae showed a negative trend over time (Supplementary Figure 6).

#### Pollution

**Fig. 4:**
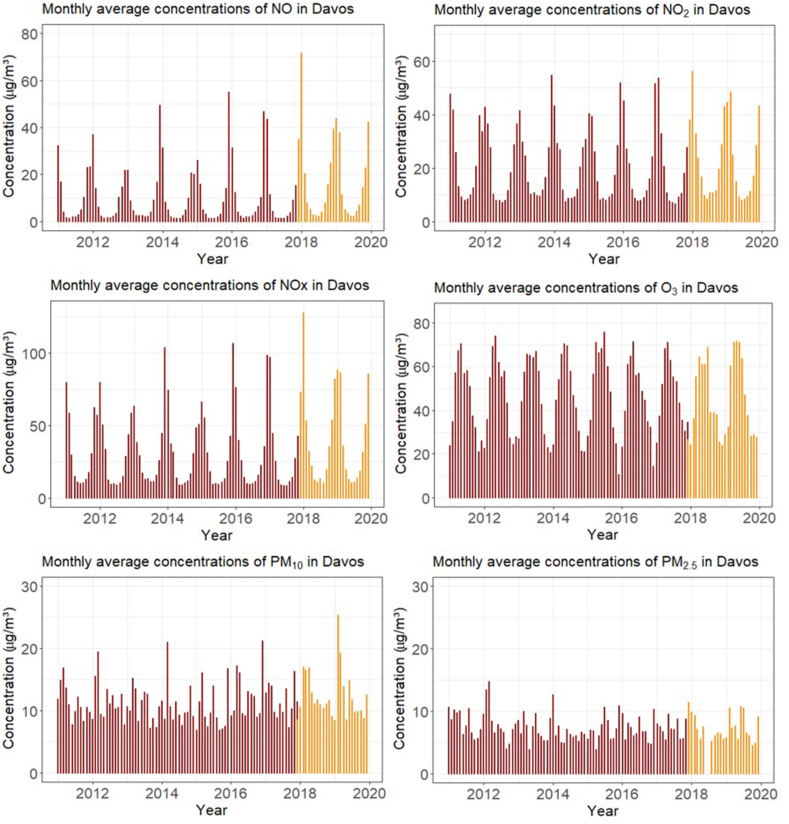
Monthly average concentrations of gaseous pollutants NO, NO_2_, NOx, O_3_, and fractions of particulate matter PM_10_ and PM_2.5_. The colors indicate the measuring stations, red for Davos Promenade DPR and orange for Davos Bubenbrunnenplatz DBP. The move had no statistical significant effect on the values (see text)

Pollution increases mainly during winter season and displays strong seasonality (Fig. 4). The monthly average concentrations were NO 13.4 ± 3.28 μg/m^3^, NOx 35.05 ± 5.88 μg/m^3^, NO_2_ 21.7 ± 2.77 μg/m^3^, PM_10_ 11.9 ± 2.18 μg/m^3^, PM_2.5_ 7.42 ± 2.22 μg/m^3^ and O_3_ 46.3 ± 4.16 μg/m^3^). Concentrations did not exceed legal limits. However, increasing trends since 2014–2015 for NOx, NO, ozone and PM_10_ were observed (Supplementary Figure 7).

#### Sensitization & atopic disease

Of the 121 children who provided blood for ISAC measurements, 51 (42%) were sensitized to at least 1 allergen component, of whom 13 (25%) were mono-sensitized and 38 (75%) were sensitized to multiple allergen components. Sensitization to aeroallergens 46 (38%) was most common, 40 (33%) of children were sensitized to grass (Phl p or Cyn d), 19 (16%) were sensitized to cat (Fel d 1) (Supplementary Table 1). Few children were sensitized to food allergens 8 (7%) and HDM 3 (2.5%).

Self-reported rates for existing allergies were 26%, for asthma 24%, for atopic dermatitis 14%, for rhinoconjunctivitis 22% and for food allergy 9%. Reported doctor-diagnosed asthma was 12%, doctor-diagnosed atopic dermatitis 15%, doctor-diagnosed rhinoconjunctivitis 18% and doctor-diagnosed food allergy 6%.

Univariate regression models demonstrated no direct correlation between exposure and senstization to any allergen component (Supplementary Table 3). Exploratory factor analysis (Principal Component Analysis) of the exposure questions from the questionnaire resulted in three factors explaining 73% of the variance: pollen exposure, parental education and indoor living conditions. Pollen exposure contains the cumulative pollen exposure variables to hazel, alder, birch, plane, grass, plantain, mugwort and ambrosia; the second factor, parental education, contains the education level of mother and father; the third factor, indoor living conditions, contains occupant density, change of bed linen, visible mold in the home, and presence of multiple detectable allergens in residential dust. Factor scores (Anderson Rubin scores) were created and directly used as a variable in the multiple logistic regression analysis. Multiple logistic regression analysis demonstrated that living close to a busy road increased the odds of being sensitized to any allergen aOR(95%CI) 2.7(1.0–7.2) (Table 2). The strength of this finding increased for sensitization together with self-reported symptoms of rhinoconjunctivitis aOR(95%CI) 4.4 (1.3–14.8) or asthma aOR(95%CI) 5.5(1.4–21). For sensitization to outdoor aeroallergens, living close to a busy road also increased the odds aOR(95%CI) 2.8(1.1–7.1). For sensitization to indoor allergens, the constructed factor indoor living conditions consisting of occupant density, frequency of bedlinen change, visible mold in the home and exposure to multiple allergens in residential dust significantly increased the odds of being sensitized to indoor allergens aOR(95%CI) 1.9 (1.1–3.2) and being sensitized plus reporting symptoms of rhinoconjunctivitis 1.9 (1.0–3.6) and asthma 2.1 (1.0–4.1).

**Table 2:** Multiple logistic regression analyses assessing the relation between exposure and any sensitization, exposure and sensitization plus doctor confirmed diagnosis of rhinoconjunctivitis, exposure and sensitization plus self-reported symptoms of rhinoconjunctivitis and exposure and sensitization plus self-reported symptoms of asthma. Sensitization was assessed as sensitized to any allergen, sensitized indoor allergens and sensitized to outdoor allergens. All models were adjusted for sex, having atopic family members and socio-economic position.

	Sensitization to any allergen		Sensitization to any allergen plus doctors diagnosis of rhinoconjunctivitis		Sensitization to any allergen plus selfreported complaints of rhinoconjunctivitis		Sensitization to any allergen plus selfreported complaints of asthma		Sensitization to indoor allergens aOR(95%CI)		Sensitization to outdoor allergens aOR(95%CI)	
	aOR(95%CI)	p	aOR(95%CI)	p	aOR(95%CI)	p	aOR(95%CI)	p	aOR(95%CI)	p	aOR(95%CI)	p
Male sex	0.7 (0.3–1.6)	0.400	0.8 (0.2–2.5)	0.705	0.67 (0.21–2.2)	0.514	0.5 (0.13–1.9)	0.323	1.2 (0.4–3.4)	0.726	0.79 (0.34–1.85)	0.590
Atopic family members	0.9 (0.4–2.1)	0.871	3.4 (0.8–14.2)	0.088	4.7 (1.1–20.8)	0.042	6.3 (1.0–38)	0.045	2.3 (0.7–7.2)	0.167	0.9 (0.4–2.1)	0.808
Socio-economic position index	0.95 (0.68–1.3)	0.770	1.0 (0.66–1.7)	0.800	1.1 (0.7–1.8)	0.639	1.1 (0.7–1.9)	0.672	1.0 (0.66–1.6)	0.924	1.0 (0.71–1.4)	0.970
Living close to a busy road	2.7 (1.0–7.2)	0.038	3.3 (1.0–11)	0.047	4.4 (1.3–14.8)	0.017	5.5 (1.4–21)	0.013	2.5 (0.8–7.6)	0.115	2.8 (1.1–7.1)	0.034
Indoor living conditions	0.87 (0.58–1.3)	0.505	1.2 (0.65–2.2)	0.554	1.9 (1.0–3.6)	0.039	2.1 (1.0–4.1)	0.044	1.9 (1.1–3.2)	0.020	1.1 (0.73–1.7)	0.633
Ever breastfed	1.4 (0.51–3.5)	0.545	1.8 (0.55–6.0)	0.326	2.4 (0.66–8.7)	0.184	2.6 (0.6–11.3)	0.195	Not included in the model		Not included in the model	

aOR = adjusted Odds Ratio, CI = Confidence Interval, p = p-value. Male sex (vs female sex), atopic family members (vs no atopic family members), socio-economic position index (continuous), living close to a busy road (vs not living close to a busy road), indoor living conditions (Anderson Rubin factor score consisting of occupant density, change of bed linen, visible mold in the home, and presence of multiple detectable allergens in residential dust), ever breastfed (vs never breastfed)

## Discussion

In this cross-sectional study we investigated the relation between environmental exposure and prevalence of allergic sensitization among school children born and resident in the Swiss alpine municipality Davos.

Sensitization to any allergen was 42% in our cohort of children aged 5–9 years and self-reported rate for any allergies was 26%. In other European birth cohorts, the prevalence of sensitization was similar or lower.^16^, ^17^, ^18^ In our study cohort, the self-reported rates for asthma was 24%, for rhinoconjunctivitis 22%, for eczema 14%, and for food allergy 9%. Compared to other studies in Western Europe and Switzerland, rates in our cohort were > three-fold higher.^2^^,^^19^^,^^20^ However, ISAAC also demonstrated large variability in prevalence and severity of atopic disease, not only between countries and cities, but also within cities.^2^

Local environmental characteristics are thought to play a crucial role in the development and prevalence of atopic disease. A Norwegian study reported an asthma prevalence of 10% in a non-polluted rural mountain area, with a comparable climate to our cohort.^21^^,^^22^ It is hypothesized that the relatively high prevalence of asthma may be associated with a high incidence of keeping animals at home combined with frequent heavy physical activity in cold weather.^22^ In Davos, alpine sports and cross-country skiing are also quite popular among school children and 43% of households reported the presence of pets inside the home. In Los Alamos located at 2194 m, a mite-free area with low outdoor pollution, asthma diagnosis was also high (15%) among children born and raised there, and possibly associated with the frequent presence of indoor pets (77%), especially cat.^23^^,^^17^

In our cohort, sensitization to grass was 33% and self-reported rhinoconjunctivitis was 22%. Several studies have reported a higher prevalence of pollen sensitization and rhinoconjunctivitis at high altitude compared to lower altitudes. In a French study comparing school children living at sea level (Marseille) and high altitude (Briancon, 1260 m a.s.l.), positive SPTs for grass pollen were found in 21.7% of children born and living at high altitude, whereas this was 8.5 % in children living at sea level.^24^ An American study including children living at 2194 m reported a 47 % prevalence of pollen sensitization.^23^ A Norwegian study reported 33% prevalence of rhinoconjunctivitis and pollen sensitization.^22^ However, pollen counts of grass and birch at these altitudes were much lower compared to sea level. It could be that the pattern of sensitization reflects the exposure to locally available allergens. Children living at altitude may spend more time outside and therefore may have a higher cumulative pollen exposure. However pollen exposure does not directly represent allergen exposure. Daily allergen release per pollen fluctuates and pollen potencies in the alpine climate may also be affected by the presence of pollen coming from long-distance geographical origin.^25^^,^^26^

Allergen exposure outside the geographical area of common residence, for example during summer holidays in places with higher pollen concentrations, may also contribute to the high prevalence of rhinoconjunctivitis. Furthermore, it is possible that populations born and raised in high altitude may have been more isolated and thus, have a more preserved genetic background, which could influence susceptibility to allergic sensitization and the development of allergic disease.^27^

House dust contains several allergens but most commonly mites, animal and fungal allergens. Detectable levels of HDM allergens were found in up to 37% of residential dust samples, but generally in low concentrations. The median concentrations of Der p 1, Der p 2 and Der f 1 were 74 ng/g, 18 ng/g and 29 ng/g, respectively. However, there was large variability among samples and some households reached concentrations as high as 4690 ng/g for Derp 1, 1622 ng/g for Der p 2 and 2457 ng/g for Der f 1. These concentrations were considered elevated in other studies as well.^28^ Another study already demonstrated no significant change of HDM concentration with increasing altitude.^29^ However, HDM sensitization in our cohort was rare (2.5%), which is line with other studies conducted at altitude.^22^^,^^23^^,^^27^^,^^30^

Apart from indoor allergen exposure, other indoor living conditions demonstrated a significant influence on sensitization to indoor allergens. We found that a combination of occupant density, frequency of bedlinen change, visible mold in the home and exposure to multiple allergens in residential dust significantly increased the odds of being sensitized to indoor allergens. Visible mold in the home has already been identified as a risk factor for developing atopic disease.^31^ Combined data from several European birth cohorts also demonstrated an association between visible mold in the home and a statistically significant increased risk for asthma and allergic rhinitis in school children.^32^^,^^33^

In our cohort, living close to a busy road was associated with increased odds OR (95%CI) for being sensitized to any allergen (3.6 (1.3–10)) and to outdoor allergens (2.8 (1.1–7.2)). These associations increased when self-reported symptoms were added to sensitization as the outcome. Several studies have reported significant associations between exposure to traffic-related air pollution (TRAP) such as PM_2.5_, PM_10_, NO_2_ and pollen sensitization or allergic respiratory disease.^34^, ^35^, ^36^ However, meta-analyses have found no clear association between traffic and allergic sensitization or childhood asthma prevalence.^11^^,^^16^^,^^37^, ^38^, ^39^ Recently, allergen specific analyses suggested increased risk for sensitization to birch, grass and cat may be related to exposure to specific pollutants.^18^

Air pollutants and pollen grains may interact through several mechanisms and the allergenic potential of pollen grains could be enhanced through contact with pollutants.^40^, ^41^, ^42^, ^43^ To accurately evaluate environmental determinants, both pollen allergen and pollutant contents should be considered.^41^

Little is known about regions with low levels of TRAP exposure and risk of allergic respiratory disease. Even though pollution levels in Davos are relatively low, we still found a significant association between living close to a busy road and allergic sensitization. Another study in a low pollution setting also demonstrated associations between living <200 m from a major road and current wheeze and allergic sensitization.^44^ These findings suggest that even in settings with relatively low pollution levels, TRAP exposure could increase the risk for allergic sensitization.

This study was designed as a cross-sectional observational study. Therefore, it is not possible to determine whether the outcome followed exposure in time and identified associations should be interpreted with this study design in mind. Furthermore, the use of questionnaires may lead to misclassification because of recall bias. Another limitation of the study is possible selection bias. Families with children with current atopic symptoms may have been more interested to participate in the study, leading to a possible overestimation of the prevalence.

Strengths of this study are the detailed assessment of sensitization using ISAAC and the extensive assessment of exposure to indoor environmental allergens as well as cumulative exposure to pollen and air pollution.

## Conclusion

In a cohort of children born and raised in an alpine environment, sensitization to aeroallergens is common (38%), especially to grass (33%) and cat (16%). House dust mite allergen was detected in up to 38% of residential dust samples, but sensitization to HDM was low (2.5%). Living close to a busy road and indoor living conditions increased the risk for allergic sensitization. In a clean and healthy alpine environment, air pollution might still be an important factor contributing to allergic sensitization.

## Abbreviations

HDM, house dust mite; TRAP, traffic related air pollution; PM10, particulate matter; NOx, nitrogen oxides; O3, ozone; IQR, inter quartile range; SD, standard deviation; ISAC, Immuno-Solid phase Allergy Chip; ISU, ISAC standardized unit; ISAAC, International Study on Asthma and Allergy in Childhood; MIA, Microarray Image Analyzer

## Funding

This study was supported by a CK-CARE Individual project grant.

## Availability of data

The data that support the findings of this study are available on reasonable request from the corresponding author. The data are not publicly available due to privacy or ethical restrictions.

## Author contributions

KF collected the data, performed the analysis and wrote the manuscript, JMM contributed data or analysis tools and performed the analysis, BR JC GP JB contributed data or analysis tools, CA and CTA conceived and designed the study. All authors read and approved the final version.

## Ethics approval and consent to participate

Written informed consent was obtained from the legal guardians of the study participants. The study was approved by the ethics committee of the University of Zürich, BASEC nr 2017–01262. All study procedures involving human participants were in accordance with the principles of the declaration of Helsinki.

## Authors’ consent for publication

Each of the authors has reviewed this paper in its submitted form and approved submission for publication of this paper to the World Allergy Organization Journal.

## Declaration of competing interest

The authors declare no competing interests.
