# Supplementary material for: Environmental exposure and sensitization patterns in a Swiss alpine pediatric cohort
Source: World Allergy Organ J. 2023 Dec 3;16(12):100847. doi: 10.1016/j.waojou.2023.100847 (PMC10701589; doi:10.1016/j.waojou.2023.100847)
Supplement: Multimedia component 1 [file mmc1.docx]

Supplementary Table 1 All components (inhalant, food, other) present on the ImmunoCAP ISAC 112, with their sensitization frequency and number of children sensitized from the study population (n=121). The most frequent sensitizations are highlighted in light grey.

| **Component** | **Source** | **Allergen Type** | **Sensitization Frequency (%)** | **Number of children (n)** |
| --- | --- | --- | --- | --- |
| Aln g 1 | Alder pollen | Inhalant | 0.8% | n=1 |
| Alt a 1 | Alternaria | Inhalant | 0.8% | n=1 |
| Alt a 6 | Alternaria | Inhalant |  | None |
| Amb a 1 | Ragweed | Inhalant |  | None |
| Art v 1 | Mugwort pollen | Inhalant | 8.3% | n=10 |
| Art v 3 | Mugwort pollen | Inhalant | 0.8% | n=1 |
| Asp f 1 | Aspergillus | Inhalant |  | None |
| Asp f 3 | Aspergillus | Inhalant |  | None |
| Asp f 6 | Aspergillus | Inhalant |  | None |
| Bet v 1 | Birch pollen | Inhalant | 3.3% | n=4 |
| Bet v 2 | Birch pollen | Inhalant | 5% | n=6 |
| Bet v 4 | Birch pollen | Inhalant |  | None |
| Bla g 1 | Cockroach | Inhalant |  | None |
| Bla g 2 | Cockroach | Inhalant |  | None |
| Bla g 5 | Cockroach | Inhalant |  | None |
| Bla g 7 | Cockroach | Inhalant | 0.8% | n=1 |
| Blo t 5 | Blomia mite | Inhalant |  | None |
| Can f 1 | Dog | Inhalant | 2.5% | n=3 |
| Can f 2 | Dog | Inhalant |  | None |
| Can f 3 | Dog | Inhalant | 1.7% | n=2 |
| Can f 5 | Dog | Inhalant |  | None |
| Che a 1 | Pigweed pollen | Inhalant | 2.5% | n=3 |
| Cla h 8 | Cladosporium | Inhalant |  | None |
| Cor a 1.01 | Hazel pollen | Inhalant | 0.8% | n=1 |
| Cry j 1 | Japanese cedar pollen | Inhalant | 0.8% | n=1 |
| Cup a 1 | Cypress pollen | Inhalant | 0.8% | n=1 |
| Cyn d 1 | Bermuda pollen | Inhalant | 16.5% | n=20 |
| Der f 1 | Dermatophagoides mite | Inhalant | 1.7% | n=2 |
| Der f 2 | Dermatophagoides mite | Inhalant | 0.8% | n=1 |
| Der p 1 | Dermatophagoides mite | Inhalant | 1.7% | n=2 |
| Der p 2 | Dermatophagoides mite | Inhalant | 0.8% | n=1 |
| Der p 10 | Dermatophagoides mite | Inhalant | 0.8% | n=1 |
| Equ c 1 | Horse | Inhalant | 3.3% | n=4 |
| Equ c 3 | Horse | Inhalant | 1.7% | n=2 |
| Fel d 1 | Cat | Inhalant | 15.7% | n=19 |
| Fel d 2 | Cat | Inhalant | 1.7% | n=2 |
| Fel d 4 | Cat | Inhalant | 4.1% | n=5 |
| Lep d 2 | Lepidoglyphus mite | Inhalant | 1.7% | n=2 |
| Mer a 1 | Mercury pollen | Inhalant | 5% | n=6 |
| Mus m 1 | Mouse | Inhalant | 2.5% | n=3 |
| Ole e 1 | Olive pollen | Inhalant | 1.7% | n=2 |
| Ole e 7 | Olive pollen | Inhalant |  | None |
| Ole e 9 | Olive pollen | Inhalant | 0.8% | n=1 |
| Par j 2 | Wall pellitory pollen | Inhalant | 0.8% | n=1 |
| Pla a 1 | Plane pollen | Inhalant | 1.7% | n=2 |
| Pla a 2 | Plane pollen | Inhalant | 2.5% | n=3 |
| Pla a 3 | Plane pollen | Inhalant | 0.8% | n=1 |
| Pla l 1 | Plantain | Inhalant | 0.8% | n=1 |
| Phl p 1 | Timothy pollen | Inhalant | 27.3% | n=33 |
| Phl p 2 | Timothy pollen | Inhalant | 7.4% | n=9 |
| Phl p 4 | Timothy pollen | Inhalant | 15.7% | n=19 |
| Phl p 5 | Timothy pollen | Inhalant | 19.8% | n=24 |
| Phl p 6 | Timothy pollen | Inhalant | 14.9% | n=18 |
| Phl p 7 | Timothy pollen | Inhalant | 4.1% | n=5 |
| Phl p 11 | Timothy pollen | Inhalant | 4.1% | n=5 |
| Phl p 12 | Timothy pollen | Inhalant | 3.3% | n=4 |
| Sal k 1 | Saltwort | Inhalant |  | None |
|  |  |  |  |  |
| Act d 1 | Kiwi | Food | 0.8% | n=1 |
| Act d 2 | Kiwi | Food | 0.8% | n=1 |
| Act d 5 | Kiwi | Food |  | None |
| Act d 8 | Kiwi | Food | 0.8% | n=1 |
| Ana o 2 | Cashew nut | Food | 1.7% | n=2 |
| Api g 1 | Celery | Food | 0.8% | n=1 |
| Ara h 1 | Peanut | Food | 1.7% | n=2 |
| Ara h 2 | Peanut | Food | 1.7% | n=2 |
| Ara h 3 | Peanut | Food | 1.7% | n=2 |
| Ara h 6 | Peanut | Food | 1.7% | n=2 |
| Ara h 8 | Peanut | Food | 0.8% | n=1 |
| Ara h 9 | Peanut | Food |  | None |
| Ber e 1 | Brazil nut | Food |  | None |
| Bos d 4 | Cow's milk | Food |  | None |
| Bos d 5 | Cow's milk | Food |  | None |
| Bos d 6 | Cow's milk & beef | Food | 0.8% | n=1 |
| Bos d 8 | Cow's milk | Food |  | None |
| Bos d lactoferrin | Cow's milk | Food | 0.8% | n=1 |
| Cor a 1.04 | Hazelnut | Food | 0.8% | n=1 |
| Cor a 8 | Hazelnut | Food | 0.8% | n=1 |
| Cor a 9 | Hazelnut | Food | 0.8% | n=1 |
| Fag e 2 | Buckwheat | Food |  | None |
| Gad c 1 | Cod | Food | 0.8% | n=1 |
| Gal d 1 | Egg white | Food | 0.8% | n=1 |
| Gal d 2 | Egg white | Food |  | None |
| Gal d 3 | Egg white | Food | 1.7% | n=2 |
| Gal d 5 | Egg yolk/chicken | Food |  | None |
| Gly m 4 | Soy | Food | 0.8% | n=1 |
| Gly m 5 | Soy | Food | 1.7% | n=2 |
| Gly m 6 | Soy | Food | 2.5% | n=3 |
| Jug r 1 | Walnut | Food | 1.7% | n=2 |
| Jug r 2 | Walnut | Food | 2.5% | n=3 |
| Jug r 3 | Walnut | Food | 0.8% | n=1 |
| Mal d 1 | Apple | Food | 0.8% | n=1 |
| Pen m 1 | Shrimp | Food | 0.8% | n=1 |
| Pen m 2 | Shrimp | Food |  | None |
| Pen m 4 | Shrimp | Food |  | None |
| Pru p 1 | Peach | Food | 0.8% | n=1 |
| Pru p 3 | Peach | Food | 0.8% | n=1 |
| Ses i 1 | Sesame | Food | 1.7% | n=2 |
| Tri a 14 | Wheat | Food |  | None |
| Tri a 19.0101 | Wheat | Food |  | None |
| Tri a aA TI | Wheat | Food |  | None |
|  |  |  |  |  |
| Ani s 1 | Anisakis | Other |  | None |
| Ani s 3 | Anisakis | Other |  | None |
| Api m 1 | Honey bee | Other | 0.8% | n=1 |
| Api m 4 | Honey bee | Other | 0.8% | n=1 |
| Hev b 1 | Latex | Other |  | None |
| Hev b 3 | Latex | Other |  | None |
| Hev b 5 | Latex | Other |  | None |
| Hev b 6.01 | Latex | Other | 0.8% | n=1 |
| Hev b 8 | Latex | Other | 5% | n=6 |
| MUXF3 | Bromelin | Other | 5.8% | n=7 |
| Pol d 5 | Paper wasp | Other | 0.8% | n=1 |
| Ves v 5 | Common wasp | Other | 3.3% | n=4 |

Supplementary Figures

Supplementary Figure 1 Households with detectable levels of allergens in residential dust samples. Der p 1 in 17% of households, Der p 2 in 37% of households, Der f 1 in 38% of households, Mus m 1 in 50% of households, Asp f 1 in 6% of households, Alt a 1 in 3% of households and Ara h 6 in 99% of households.

Supplementary Figure 2 Indoor allergen concentration of Ara h 6 (ng/mg) measured in residential dust.


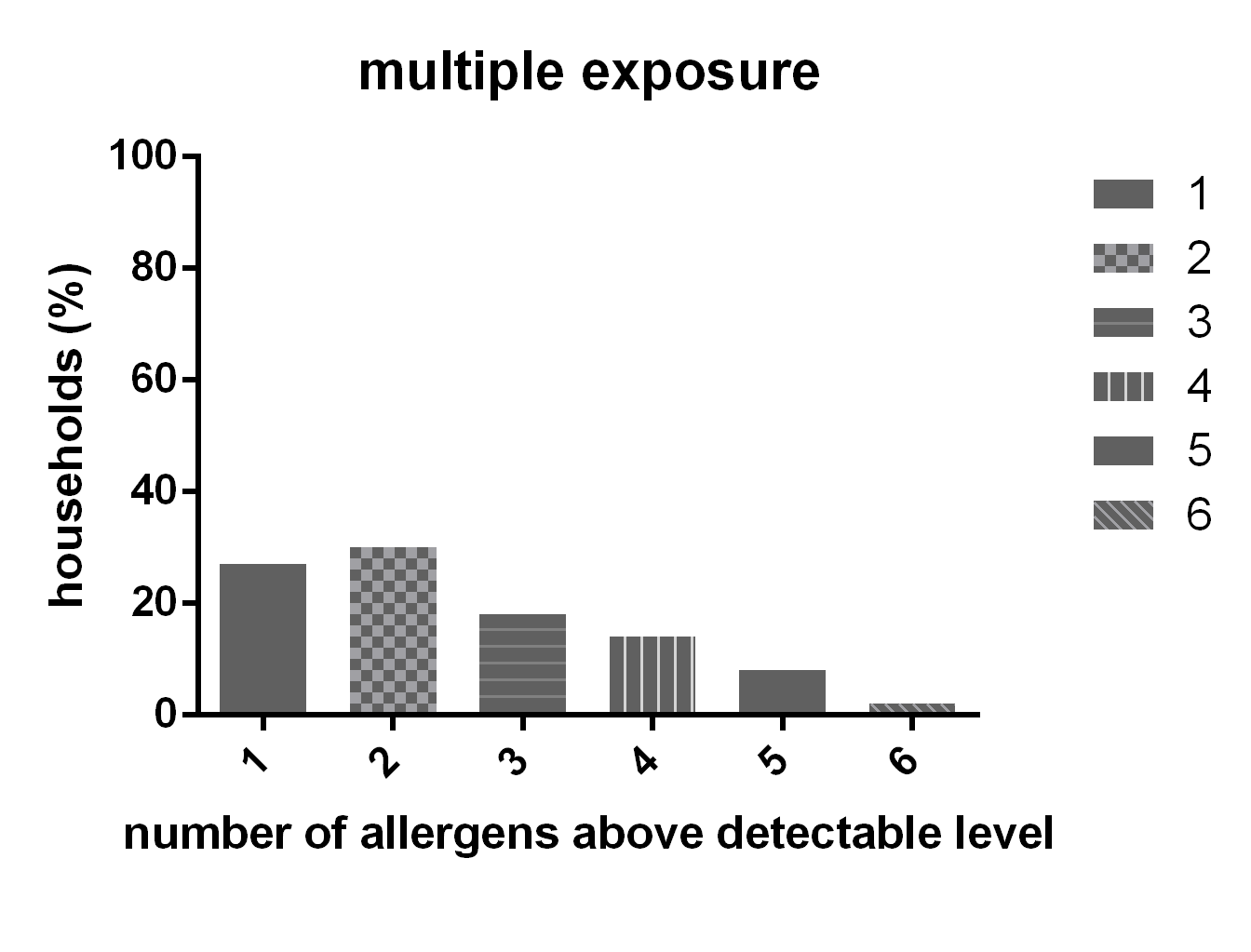


Supplementary Figure 3 Exposure to multiple allergens within a single residential dust sample. In 27% of households only peanut allergen Ara h 6 was detected, in 31% of households 2 different allergens were detected, in 18% of households 3 different allergens, in 14% of households 4 different allergens, in 8% of households 5 different allergens, in 2% of households 6 different allergens were detected.


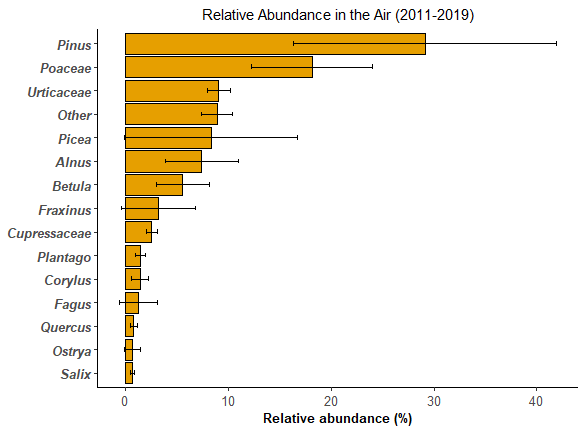


Supplementary Figure 4 Relative abundance of pollen in Davos during the study period obtained from the pollen station of Davos-Wolfgang (DPS), located at coordinates 46.828036° N and 9.854311° E, with an elevation of 1587 meters above sea level. The intake hole for the pollen trap was located at 16.50 meters’ height (on 15.00 m-roof).


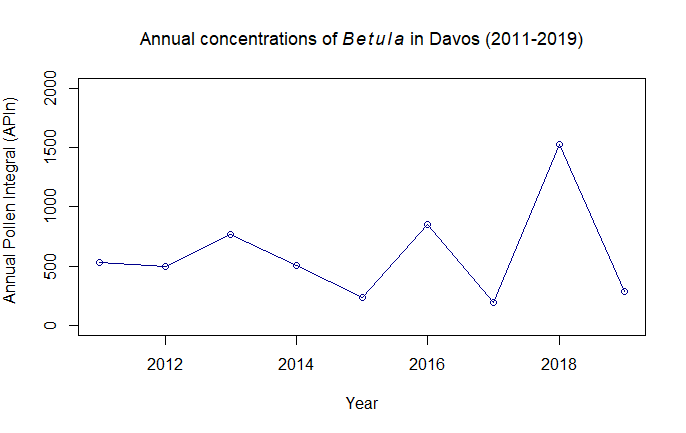

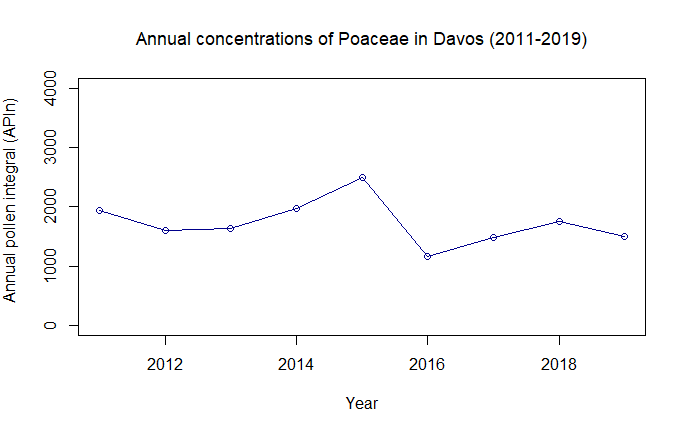

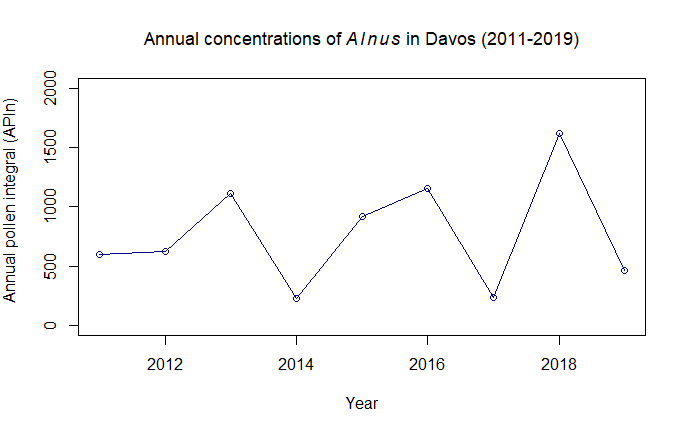

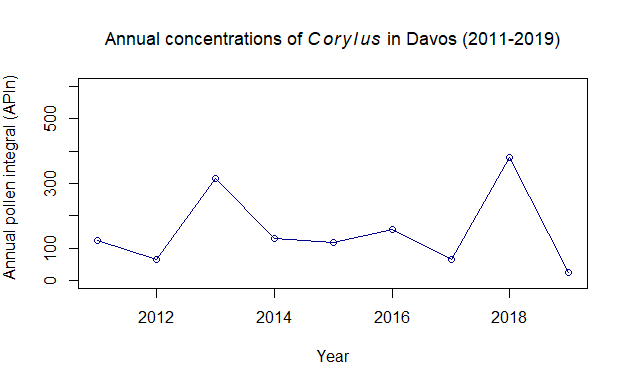

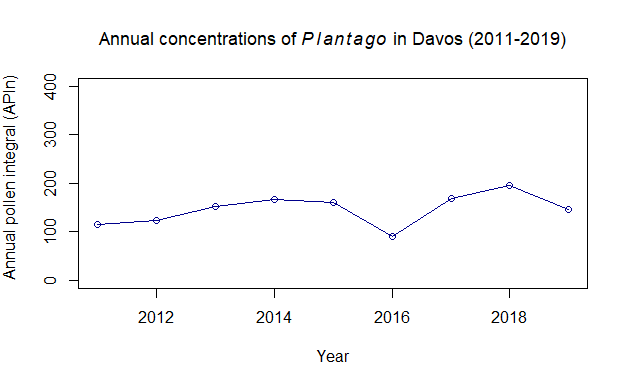


Supplementary Figure 5 Annual pollen concentrations of *Betula*, Poaceae*,* *Alnus, Corylus* and *Plantago* (all pollen with and APIn > 100 pollen* day/m^3^) in Davos from 2011 to 2019.


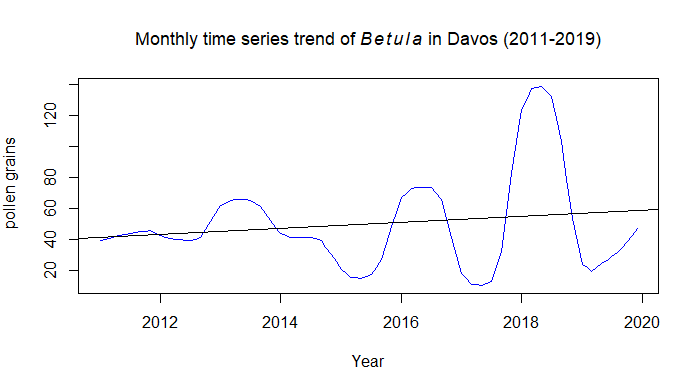

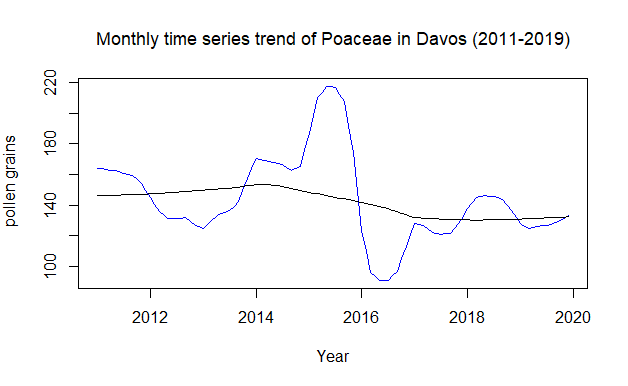

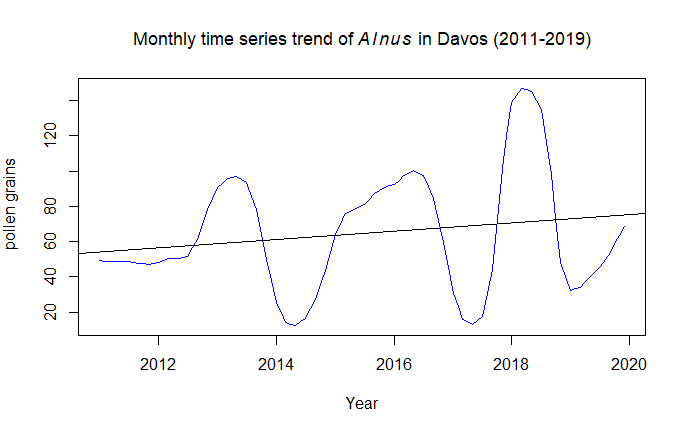

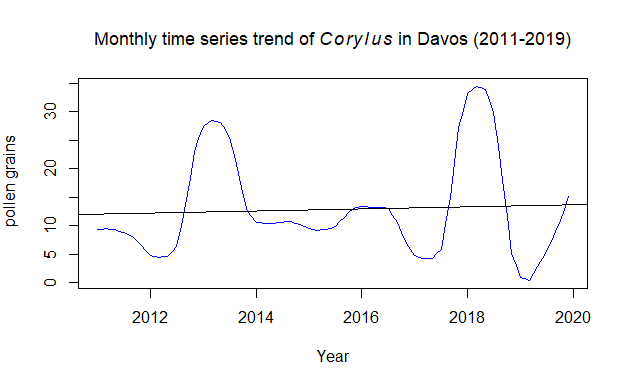

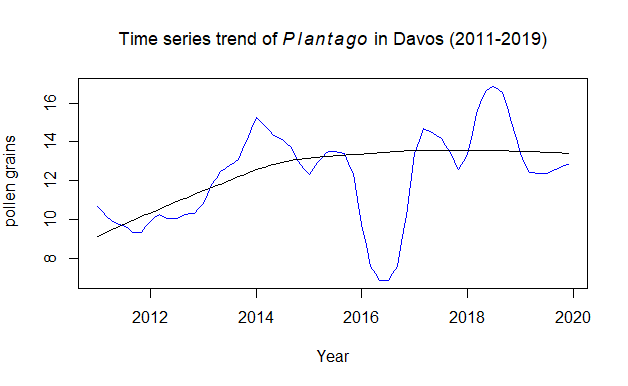


Supplementary Figure 6 Monthly time series trends of *Betula*, Poaceae, *Alnus, Corylus* and *Plantago* (all pollen with and APIn > 100 pollen* day/m^3^) in Davos from 2011 to 2019.


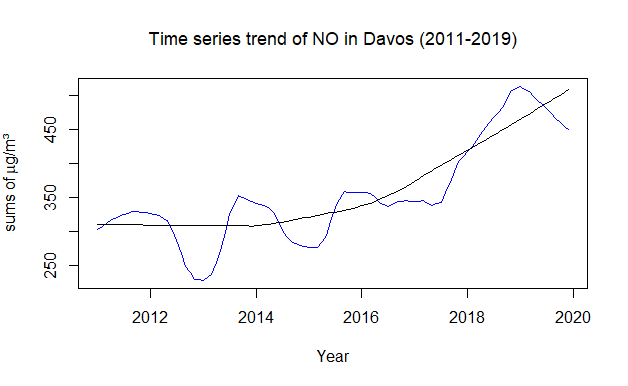

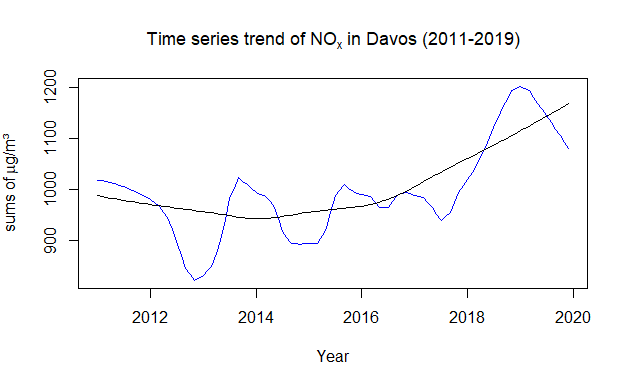

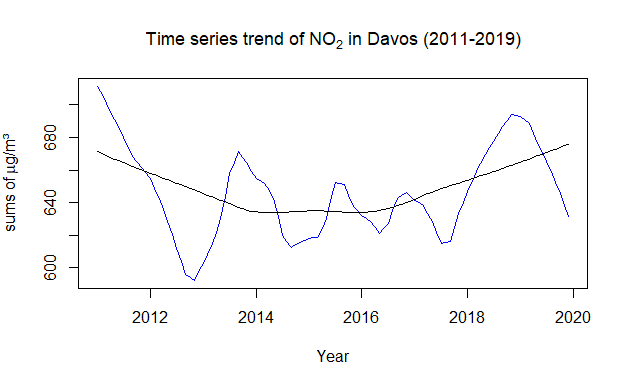


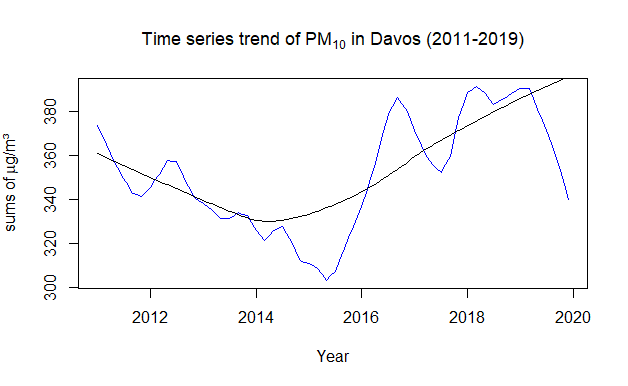


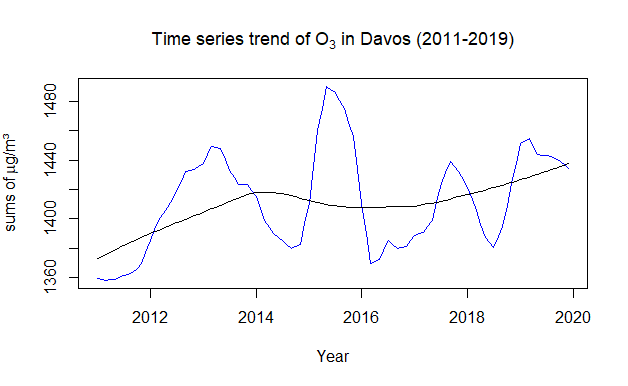


Supplementary Figure 7 Trends in monthly sums for NO_x_, NO_2_, NO, PM_10_ and O_3_ concentrations for Davos during the period 2011-2019 (DPR + DBP).
